# Embroidered Transmission Lines with Conductive Yarns: Challenges, Modeling, Fabrication, and Experimental Performance Assessment

**DOI:** 10.3390/s24216961

**Published:** 2024-10-30

**Authors:** Chrysanthi Angelaki, Aris Tsolis, Sofia Bakogianni, Antonis A. Alexandridis

**Affiliations:** Institute of Informatics and Telecommunications, NCSR “Demokritos”, 15341 Athens, Greece; chr.angelaki@iit.demokritos.gr (C.A.); sof.bakogianni@iit.demokritos.gr (S.B.); aalex@iit.demokritos.gr (A.A.A.)

**Keywords:** conductive yarns, embroidered textile transmission lines, measurements, effective conductivity

## Abstract

This paper presents an enhanced measurement technique for evaluating embroidered transmission lines (TLs), based on a TL characterization method. The evaluation metric is the “pure” losses of the embroidered TL excluding mismatch losses. Enhanced mechanical stability and removability of embroidered samples under a test is supported by a specially designed measurement setup. Losses are used to find the effective conductivity of each embroidery pattern. Various embroidered samples are fabricated, measured, and evaluated. The repeatability of measurements and fabrication are analyzed and assessed, resulting in average deviations of 0.5 dB and 0.7 dB, respectively. A comparative evaluation of two different yarns of low and high conductivity is presented. Single and double stitching patterns for each yarn are manufactured with stitch densities of 1–7 lines/mm. For interconnection with SMA connectors, a conductive fabric contact (CFC) was selected as the finish of the TL, as a more practical interface instead of direct yarn contact (YC). The analysis of the measurements proved useful findings, such as an increase in the stitch density or the amount of yarn used does not always improve the performance; the use of double stitching greatly improves low-performance stitch densities; the effective conductivity of embroidery patterns changes with frequency; the YC interface yields more losses for medium stitch densities, but for higher stich densities, it presents an improved performance compared with the CFC interconnection.

## 1. Introduction

The evolution of textile electronics, antennas, and TLs has rapidly grown over the last two decades, with various research achievements [1]. As extensively mentioned and analyzed in [2], an important category of textile antennas/electronics are the ones fabricated via embroidery techniques, mostly using conductive yarns [3,4] to implement the main conductive parts. The use of conductive threads provides flexibility and strength so as not to be broken by the high tensions applied in both the fabrication and operation phases of the textile devices [5].

The use of conductive yarns offers great potential for wearable textile antennas/electronics to be easily embedded into clothes [6,7,8] presenting comparable performance to conventional conductive materials, such as copper [9]. Also, in comparison with conductive cloths they are more flexible and recoverable [9] from stretching [10], bending [11,12] washing [13], etc. [14]. Moreover, they comply with mass production requirements.

Focusing on the area of wearable sensors, the use of embroidered TLs or general embroidery structures constitutes an open research topic covering the following application areas:

(a) The design and fabrication of embroidered TLs, instead of using the rigid conventional ones (e.g., coaxial cables). The use of TLs aims to interconnect the sensors placed on the body. The embroidered textile nature of the lines facilitates their integration into clothes and enables the flexibility of the user [2].
(b) The design and fabrication of embroidered antennas acting as sensors. Such work is described in [15], where embroidered strain sensors are presented. Commercial embroidered force-strength sensors are provided in [16]. A theoretical analysis and implementation of wearable sensors and embroidered sensors is described in [17].
(c) The design and fabrication of embroidered antennas operating as Rx/Tx antennas in a wearable system for the transmission of information from on-body sensors to an off-body monitor [4].

Despite its significant potential, the field of embroidered textile electronics is facing the following main challenges [18,19]:

(1) The selection of the most appropriate conductive yarn in terms of conductivity, stress and thermal tolerance, mechanical flexibility, and size;
(2) The effective evaluation of specific yarns and embroidery patterns designed for special applications;
(3) The selection of appropriate embroidery stitch densities [20,21], types of embroidery patterns and stitch directions so as to follow the current flow [22], so as to maximize efficiency;
(4) The design and fabrication of appropriate interface as means to interconnect embroidery patterns with other conductive textile or rigid electronics so as to avoid possible intolerances of conventional direct soldering [23,24];
(5) The embroidery design [25] and fabrication procedure [26].

### 1.1. Facing the Challenges of Embroidered Conductive Yarns

The above challenges have been addressed by various research works through several scientific approaches.

As for the first challenge (*yarn selection*), various works considered comparing different embroidery conductive yarns so as to conclude to most appropriate one. In [27] Amberstrand and copper yarns have been compared in terms of manufacture and performance via fabricated dipoles. The copper yarn was created using thin copper twisted filaments. In [28], various yarns, such as Amberstrand and Liberator, with different filaments and properties were compared in terms of fabrication accuracy and TL losses. The lowest number of filaments (threads) in yarn led to a more stretchable, tolerant, and efficient structure. In [29], steel yarns were compared with conductive cloths in terms of antenna performance and provided comparable results.

Considering this challenge for the current paper, two different conductive yarns were used and studied: Shieldex with linear resistance of about 3000 Ω/m and Elitex with much lower linear resistance of about 20 Ω/m. The results suggest that the Elitex could result in higher performance results. The Shieldex yarn is softer in terms of manipulation and fabrication compared to the Elitex one, which results in smoother patterns. Also, for double-stitched patterns, the Shieldex yarn significantly improves their EM performance and results in smoother patterns than the Elitex yarn. Through the study presented in this paper, the pros and cons of both yarns are unveiled.

As for the second challenge (*evaluation technique*), significant research works have been published. The evaluation of the embroidered yarns and their possible patterns has been conducted in a variety of ways, such as TL [30] and antenna performance tests. In [31], a TL technique for evaluating embroidered patterns as inhomogeneous structures was presented. The technique uses the S-parameters as an input to calculate characteristic impedance and conductivity. Also, a TL technique using the forward loss factor (FLF) as a means of evaluation is presented in [32], where embroidered patterns are evaluated correspondingly. Both methods are proposed in [33], where e-fiber embroidered yarn was analytically characterized and evaluated (using copper for comparison) via the TL technique (insertion loss) and patch antenna array performance (gain, efficiency). Also, in [34], the RFID antenna performance method was used to evaluate embroidered antennas. Finally, in terms of the antenna performance method in [35], the Shieldex yarn was evaluated for a stitch density of 4 lines/mm in terms of loss, gain, and effective conductivity. The effective conductivity found is close to and like those found from the analysis presented in the next sections for the specific yarn and stitch density.

Given this challenge, it should be stated that in our work, a new enhanced technique for evaluating textile TLs with different embroidery characteristics (patterns, yarns, stitching, etc.) has been adopted. The evaluation metric parameter used by this technique is the losses (pure losses) of the embroidered TL line, filtering out the mismatch losses at the ports (in/out) of the line. The corresponding measurements of this method are supported by a specially designed measurement setup arrangement (jig) that ensures that every embroidered sample will be measured as a part of a microstrip line that has the same ground plane, substrate and coaxial connectors. This provides a relatively stable and repeatable measurement technique with the average difference in the loss measurements between the embroidered samples being approximately 0.5 dB. A measurement campaign of an extensive number of embroidered samples led us to estimate the effective conductivity corresponding to various embroidery patterns. The presented evaluation measurements method contributes to the improvement of methods used in other similar works. Most of previous research papers comply by evaluating the embroidered lines by measuring the insertion loss (S_21_) which includes the effect of reflected powers (mismatch losses at the line ports) and the FLF factor, which is more suitable for stable, soldered lines and not very appropriate for removable patterns.

As for the third challenge (*embroidery parameters*), numerous research works have been published that present a variety of performances, applications, and significances of different stitch densities, patterns, stitch directions etc. In [36], the effect of stitch density and of double stitching (a conductive pattern over a conductive pattern) are analyzed via experiments, leading to 0.1 mm manufacture surface precision. A thorough analysis of the effect of the embroidered pattern on the antenna (RFID dipole tag) performance was presented in [37]. The sewed patterns are zigzagging and parallel to the RFID dipole tag. The zig-zag pattern yielded an improved antenna performance. Also, the effect of different embroidered ground planes patterns on a patch antenna performance are investigated in [38], and numerous findings are concluded. Additionally, graded and non-graded patterns are compared in [39], proving the importance of creating graded embroidered patterns for specific applications, such as origami and/or bent antennas or for specific antenna structures [40]. The graded part of the pattern is created by reducing the stitch density, i.e., from 7 lines/mm down to 4 lines/mm, resulting in enhanced flexibility in bending without significantly reducing the performance. Similar reasoning regarding variations in stitch density and their effect on antenna performance has been presented in [41], where the density is decreased at parts of the antenna with a narrower conductive width. The effect of the running pitch for specific stitch density is described in [42]. The density of threads and pattern orientation in combination with thread tension during stitching affect the conductivity of the embroidered pattern according to [43]. Finally, the effect and the use of a meshed embroidered pattern instead of a fully filled or uniform pattern is examined in terms of performance and cost trade-off as well in [44]. The efficiency reduces by two thirds compared to full copper filled antenna, but the cost reduces as well.

Considering this challenge, the stitch directions that have been employed for all patterns and yarns in our work are parallel to the flow of the current on the TL. Seven stitch densities have been examined for each yarn (Shieldex, Elitex) (1 line/mm–7 lines/mm). The evaluation of all the stitch densities provided detailed information of the effects of this parameter on the performance. Moreover, it yielded valuable insights into how beneficial, or not, the increase in the stitch density of each yarn would be. Also, double-stitching patterns have been evaluated and compared with single stitching patterns. According to theory, this should be beneficial, and according to our research, this is the case for both yarns, but not with a similar effect. Lastly, it has been found that double stitching is more beneficial when low stitch densities and low-performance yarns, like Shieldex, are employed.

As for the fourth challenge (*interconnection interface*), various works have presented potential solutions. A technique to make the interconnection between the coaxial connector (SMA) more flexible and an embroidered pattern for ink soldering has been examined in [45]. The conventional soldering presented a worse performance than the ink flexible soldering. The effect and comparison the of embroidered and conductive epoxy interconnection of IC tags are revealed in [46]. Embroidered IC tags with copper tape as IC interconnectors are presented in [47]. Also, snap-on buttons are proposed as interface means with conductive patterns in [48] showing very good performance. Finally, an interconnection between the IC module and conductive yarn has been achieved by knitting [49]. The majority of the research works use conventional soldering, since their objective is to compare other performance parameters, and employ the standard interface.

Considering this challenge and within the scope of our work, all the studied embroidered patterns employ a small piece of a highly conductive cloth (Shieldit) at their open ends. The embroidered part of the TLs partially overlaps with the conductive cloth, which serves as an appropriate interface (realistic and stable) for wearable electronics, and as a means for interconnecting with a coaxial connector (SMA). This provides a more realistic solution to wearable textile electronics/antennas by ensuring uniformity and stability for measurements of all stitch densities and patterns. In practical applications, the proposed solution would eliminate the need for soldering of the SMA directly to the heat-sensitive conductive yarn, stability, and uniformity of current [19]. Most research papers have not examined this so far. However, to compare the interconnection techniques used thus far, direct contact (yarn contact) samples have been compared with the respective conductive interface (conductive fabric contact) samples. The samples with direct yarn contact have been fabricated for single and double stitching and for stitch densities that exhibit remarkable results for the interface case (with conductive cloth) and for the highest examined stitch density. The latter, according to [19], leads to the best performance, which has not been the case for all the yarns examined in the current paper.

As for the fifth challenge (*fabrication procedure*), several studies have presented various similar techniques for embroidered design and manufacturing [50]. It must be noted that most of the previous works consider fabrication issues (e.g., running pitch etc.). Two different stitch techniques have been presented in [51], where the back stitch and satin stitch techniques are compared, with the latter offering structural stability. Also, a multilayer embroidering technique was proposed in [52]. An analytical embroidery fabrication method which employs a laser cutting method is described in [53]. The main aim of the fabrication challenge is the smoothness of the embroidered pattern.

It must be noted that each embroidery machine has its own sensitivity and its own design environment, and according to [19], adjustments need to be made. Regarding our work, the dimensions of the microstrip line designs (EM simulation models) have been modified, considering each yarn’s thickness. The speed of the machine has been settled to a minimum. This challenge does not adhere to specific, strict rules; however, if one were to be established, it would be to avoid breaking the yarns and to embroider as smoothly as possible.

Facing all the presented challenges, our research determined the following specific findings (practical insights):

- The proposed measurement technique provides a stable and repeatable environment.
- The design settings and fabrication of embroidered patterns for TLs can be acceptable in terms of manufacturing repeatability when adjustments of the design CAD are made, considering the yarn thickness and speed (tolerance) of the embroidery machine.
- Increasing the stitch density will not always improve performance.
- Low performance stitch densities can be greatly improved by using double-stitching patterns.
- Double stitching is more beneficial for low-performance yarns and deteriorates high-performance yarns at high frequencies (>3 GHz).
- Effective conductivity of embroidery patterns is frequency-dependent, with high degradation after 3 GHz.
- Using more meters of yarn is not always advantageous.
- Direct contact (yarn contact) feeding yields more losses than the interface contact (conductive fabric contact) for medium stitch densities and improved performance at higher stitch densities.

The objective of our study is to derive practical insights that can significantly impact the implementation of embroidered TLs, as well as embroidered antennas and other embroidered/textile microwave components. Our research is a systematic investigation of embroidered, textile microstrip TLs. This study utilized two distinct types of conductive yarns, including the use of seven different stitch densities for embroidered sample preparation. Additionally, we conducted investigations on double-stitched samples. As we progressed through our experiments, it became clear that there is a significant need to modify the microstrip line design to align with the conductive yarn and stitch density, encompassing adjustments in dimensions and feeding techniques. Our observations provide profound insights concerning embroidery design modeling and fabrication, impacting not only the design and fabrication of embroidered TLs but also extending to embroidered textile antennas.

### 1.2. Embroidered Lines Evaluation Process and Sections Briefing

The paper is structured as follows: Section 2 describes the methodology and materials used, Section 3 presents the embroidery design and implementation procedure, Section 4 presents the measurement results of embroidered patterns in details, Section 5 describes the comparison between different interfaces, and, finally, in Section 6 conclusions are drawn.

**Figure 1:**
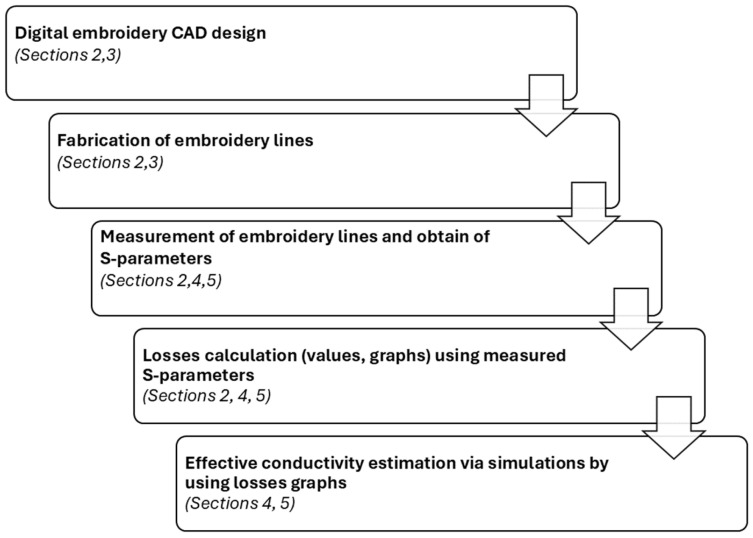
Evaluation process of embroidered transmission lines using the proposed technique.

In Figure 1, the analyzed evaluation process is depicted as a whole, including the main research topics on the embroidered TL that this work deals with (design, fabrication, measurements, evaluation of embroidery patterns, and conductivity estimation) along with the corresponding sections of the paper.

## 2. Methodology and Materials

In this section, we will outline the methodology that has been employed for measuring the fabricated samples and processing the measurement results to enable a rigorous evaluation of the embroidery parameters under investigation. Additionally, we will introduce the materials utilized for the fabrication of the examined embroidered TL samples.

### 2.1. Measurements Setup and Loss Calculation

A conventional microstrip line was designed and simulated at first as a basic reference design model. Based on this model, the pattern of an embroidered microstrip line was designed and fabricated with conductive yarn on a denim base fabric.

**Figure 2:**
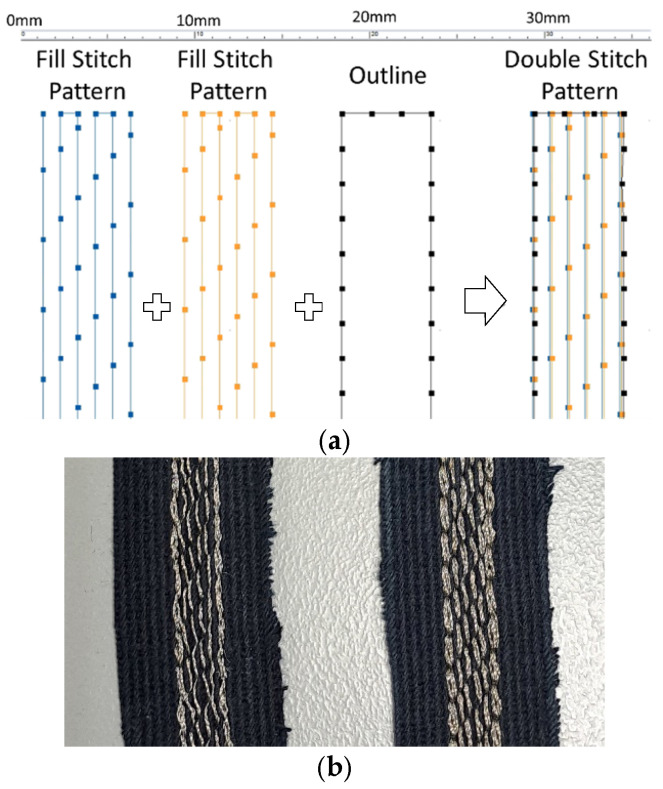
(**a**) Double-stitch (ds) pattern formation. (**b**) Close-up of an embroidered microstrip sample with density 1 line/mm, with single and double stitching, respectively.

To investigate the impact of embroidery stitch density on TL performance, we examined seven different stitch densities, denoted as di (i = 1, 2, …, 7), ranging from 1 to 7 lines/mm. Here, 7 lines/mm represents the maximum density supported by the utilized embroidery machine (Brother PR670E, Brother Sewing Machines Europe GmbH, Bad Vilbel, Germany). In parallel, we created a second set of samples to study the effects of double stitching (ds) on the performance of the TLs, denoted as di (ds). More specifically, the double stitching embroidery pattern for a specific density consists of the same fill pattern stitched twice and the outline stitched only once (Figure 2). To minimize fabrication and measurement uncertainties, two samples (sample “a” and sample “b”) were fabricated for each density, labeled as di_a and di_b. Moreover, two conductive yarns were studied, namely the Shieldex [54] and Elitex [55] yarns. In total, 28 samples were prepared for each type of yarn under study. Table 1 provides a detailed notation guide for reference throughout this paper, with additional info for the total number of stitches for each sample. While Section IV (C) will present fabrication repeatability measurements for similar samples (di_a and di_b) and measurement procedure repeatability measurements for twice repeated measurements of the same sample. All other measurement results will refer to the average of the measured values between two samples for each density. Therefore, the results for a di sample corresponds to the average of the derived measurement values of samples di_a and di_b, both embroidered with the same pattern at density i.

**Figure 3:**
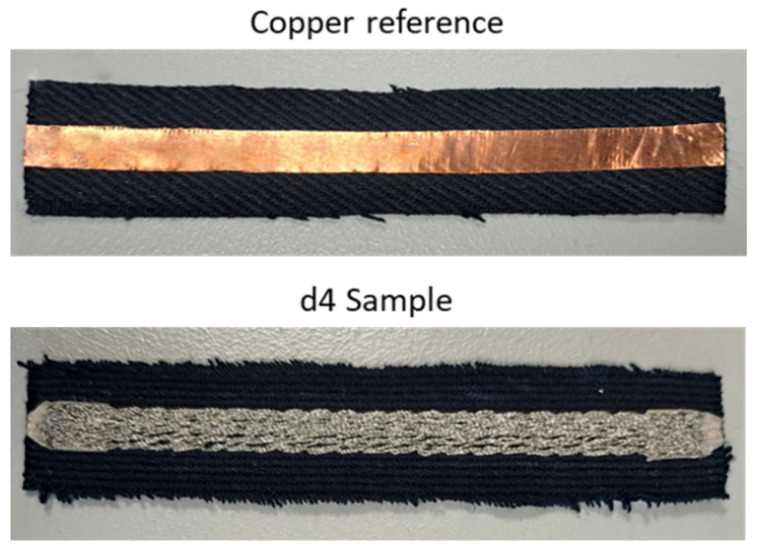
Copper reference sample and d4 sample.

Furthermore, we created a reference TL using copper tape on a denim base fabric (denoted as “copper”), which was measured on the same substrate as the embroidered samples (Figure 3). The length and width of the copper TL were 100 mm and 6 mm, respectively.

**Figure 4:**
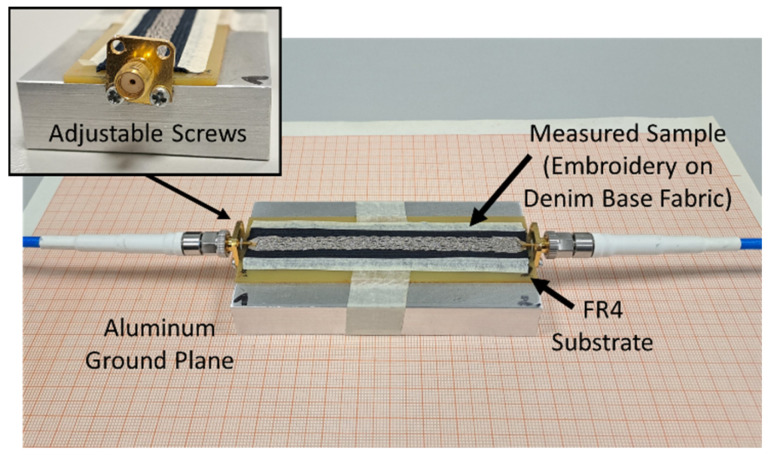
Measurement set-up of the proposed method.

With the intention of reducing the number of construction differences between the samples, a common, rigid dielectric substrate (FR4) was used for assembling and measuring all the TL samples. For the same reason, no soldering was applied for the connection of the SMA connectors to the ends of the embroidered microstrips. Instead of soldering, a specific contact method was adopted using a specially designed jig for the measurements (Figure 4). The measurement set-up (jig) is comprised of a 1.6 mm-thick FR4 substrate (*ε_r_* = 4.4, *tan* *δ* = 0.02) (with removed top and bottom copper cladding), and a solid aluminum base (100 mm × 15.3 mm × 50 mm) acting as the ground plane. The SMA connectors were attached with adjustable screws to the aluminum ground plane. The connection of the SMA pin with the measured TL could be achieved by touching the textile TL. The required pressure can be achieved by properly adjusting the screws. The two ports of a vector network analyzer (VNA) were connected to the SMA connectors of the measurement jig in order to measure the S-parameters of each embroidered sample. A paper tape was used for the attachment of the FR4 substrate to the aluminum ground plane. Paper tape was also used to secure the measured sample on the substrate, as seen in Figure 4.

**Figure 5:**
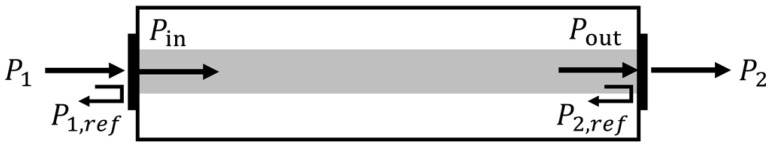
A two-port network of a microstrip line.

The S-parameters of each microstrip line were obtained by individually positioning a single sample on the common substrate. These measurements data provided the transmission and reflection characteristics of the embroidered microstrip lines. Based on the two-port network model of a TL (Figure 5), the losses of the embroidered TL can be expressed by the ratio L of Pin and Pout (Equation (1)). Considering the reflection coefficients at input and output port of the TL (Γ_in_ and Γ_out_, respectively), we can calculate the line losses neglecting the reflections at input/output ports (Equation (4)). Thus, the derived losses of the various embroidery line samples are independent of the reflected power at each port ($P_{1,ref},\;P_{2,ref}$).

(1) $$L=\frac{P_{in}}{P_{out}},$$

(2) $$P_{in}=P_{1}\left(1-{\left|\Gamma_{in}\right|}^{2}\right),$$

(3) $$P_{2}=P_{out}\left(1-{\left|\Gamma_{out}\right|}^{2}\right),$$

From Equations (1), (2) and (3) we derive the following:

(4) $$L=\frac{P_{1}}{P_{2}}\left(1-{\left|\Gamma_{in}\right|}^{2}\right)\left(1-{\left|\Gamma_{out}\right|}^{2}\right)$$

The Equation (4) can be transformed using the measured S-parameters for each embroidered line sample in the following Equation (5):

(5) $$L=\frac{\left(1-{\left|S_{11}\right|}^{2}\right)\left(1-{\left|S_{22}\right|}^{2}\right)}{{{|S}_{21}|}^{2}}$$

Equation (5) has been used to quantitatively assess the losses of each microstrip line sample, thus contributing to a comprehensive evaluation of different embroidery stitch densities and the type of conductive yarns used.

### 2.2. Conductive Yarns and Materials Used

The studied textile microstrip TLs consist of conductive threads embroidered on a denim base fabric using a computerized embroidery machine (Brother PR670E). Two conductive yarns were studied, namely Shieldex silver-plated nylon 66 yarn 117/17 dtex 2-ply (117 dtex/17 filaments) (linear resistance <30 Ω/cm = 3000 Ω/m) and Elitex Art.235/f34_PA/Ag (235 dtex/34 filaments) (linear resistance ~20 Ω/m). The specifications of each yarn are presented in Table 2. These yarns have been selected as two opposites in terms of properties. The Elitex could result in higher performance results. The Shieldex yarn is softer in terms of manipulation and fabrication compared to the Elitex one, which will result in smoother patterns. For the rest of this paper, the yarns will be referred to as Shieldex and Elitex, respectively.

For the base fabric of the embroidered microstrip TLs, a 0.7 mm-thick denim fabric was chosen. Denim, known for its structural resilience, is able to withstand multiple needle punches even at high stitch densities or with double stitching. Its ability to endure the high tensions introduced by conductive yarn stitches made it an ideal choice, despite its higher losses compared to the other base fabrics presented in the literature (e.g., organza) [16].

The permittivity (ε_r_) and loss tangent (tan⁡δ) of denim fabric used in our study was initially measured using a split-post dielectric resonator [56] at 2.4 GHz. The measured permittivity and tan⁡δ were ε_r_ = 1.92 and tan⁡δ=0.07, respectively. However, the tan⁡δ was subsequently adjusted based on the measured loss of the reference copper microstrip line. Initially, the simulated and measured results did not completely align at 2.4 GHz. For this reason, a more accurate value was obtained through parametric simulations. For tan⁡δ=0.035, the measured and simulated loss curves almost coincide up to 3 GHz. Furthermore, it was noted that the losses appeared to be overestimated at higher frequencies (3–4 GHz) using the simulation results compared to the measurements. This discrepancy was ascribed to the frequency-dispersion characteristic of the denim tan⁡δ, which was finally assumed to be constant and equal to 0.035 up to 2.5 GHz and then to decrease linearly with frequency, reaching 0.025 at 4 GHz.

As a more realistic approach and to ensure effective connections at the open ends of the TLs, conductive fabric (Shieldit Super Fabric [57]) was used. The fabric ensures smooth current flow across the width of the microstrip lines, which is challenging, especially in designs with low stitch density, as described in the following section.

## 3. Embroidery Design and Implementation

The following section will cover the modeling process of the embroidered TLs, as well as the experimental layout. The layout facilitated fast and accurate measurements of a large number of samples while minimizing the parameters that increase the measurement uncertainty. Moreover, the manufacturing repeatability was investigated.

**Figure 6:**
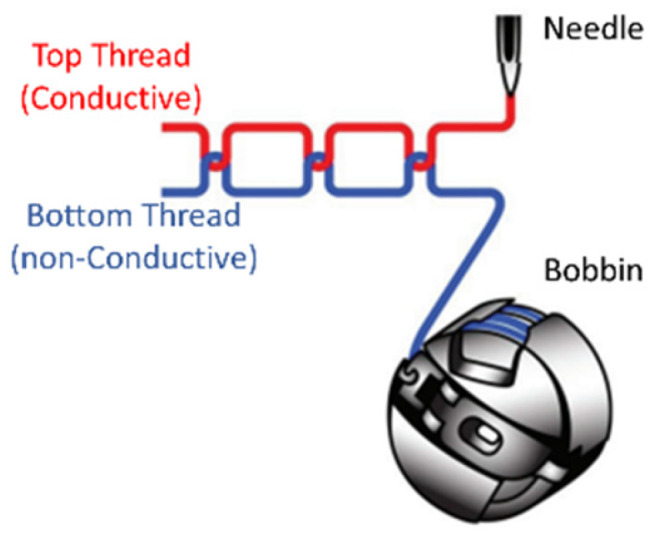
Lockstitch formation and thread positions.

The embroidery machine used in our study employs the lockstitch technique to attach the embroidery thread onto the base fabric (Figure 6). In the process of embroidering the microstrip samples, conductive yarn was placed on the top thread position. While some studies suggest that placing the conductive yarn on the bottom (bobbin) position is favorable [39,58], we selected the top placement. This decision was based on the fact that the top part of the embroidery corresponds to the design with higher accuracy and precision compared to the bottom part [59]. A strong polyester thread was used at the bottom position. The polyester bobbin yarn provides a resilient base underneath the conductive pattern, which minimizes the thread pulling and snapping of the upper, stiff conductive yarn.

In terms of the experimental embroidery design, a fill-stitch pattern with stitches aligned parallel to the direction of current flow on the TL was applied to all embroidered lines. This stitch direction was intentionally chosen in order to minimize surface resistance and insertion loss, as proposed in previous studies [21,37,58]. The stitch length for filling each line’s area was automatically set to 4 mm by the embroidery machine software (PE-DESIGN PLUS 2). An additional running stitch outline, with a stitch length equal to 2 mm, was incorporated to enhance design accuracy. The usual stitch length that was used in other works, referred to in the Introduction, is between 2 mm and 4 mm for inner and outer embroidered patterns.

When designing the embroidered lines on PE-DESIGN PLUS 2, Brother’s Personal Embroidery Design Software package, the following adjustments were made, taking into consideration the practical limitations of the embroidery procedure and the thread’s thickness: The dimensions of the designs were modified compared to the reference copper line, with the width set to 5 mm to accommodate the yarn thickness. The length of the line was increased to 104 mm to account for fabric shrinkage caused by thread pulling. To prevent thread breakage attributed to the stiff conductive filaments, the embroidery machine’s speed was set to the lowest setting, i.e., 400 stitches/minute.

**Figure 7:**
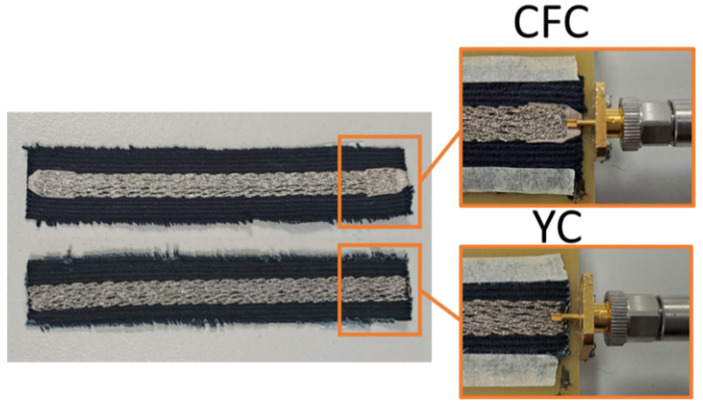
Conductive fabric feeding (CFC) and yarn contact (YC) feeding techniques.

As briefly mentioned in previous section by properly fixing the screws of the supporting jig, the appropriate pressure to the contact point of the coaxial SMA connectors pin to the conductive embroidery was applied. In this way a stable attachment of the SMA connectors to the embroidered samples without soldering was ensured. However, the connection between adjacent parallel stitches was not always guaranteed, particularly in embroidered lines with low stitch densities. This uncertainty raised concerns about the uniformity of current flow across the entire width of the microstrip line ends. To address this issue, we affixed a small piece of conductive fabric (dimensions: L = 4 mm, W = 5 mm) to each open end of the microstrip embroidered sample at the beginning of the embroidery process. The embroidery partially overlapped with the inner half of the conductive fabric (Figure 7), allowing smooth current flow to all parallel stitches, even if they were not in direct contact with the connector’s pin. For the rest of this paper, this feeding technique will be referred to as “Conductive Fabric Contact” (CFC feeding).

Our experiments revealed that this feeding technique performed well with sparsely stitched microstrips (up to 4 lines/mm). However, it led to increased losses when stitch densities exceeded 4 lines/mm. Consequently, we undertook the fabrication of additional fully embroidered samples, focusing on two specific densities: 4 lines/mm and 7 lines/mm. For these samples, we opted for direct contact between the SMA connector’s pin and the embroidered conductive yarns. The dense stitch pattern ensured uniform current flow across the entire width of the microstrip ends. We call this feeding technique “yarn contact” (YC feeding), and samples that employ this feeding technique are denoted with the suffix “YC”.

## 4. Effect of Embroidery Density and Yarn Properties

In this section, we investigate the influence of embroidery density and yarn characteristics on the performance of embroidered microstrip TLs. Initially, we present the measured results of microstrip lines utilizing single stitching, followed by the results for microstrips with double stitching. The fabrication and measurement repeatability is examined in terms of RMS difference using the parameter L[dB]. Additionally, we introduce the estimated effective conductivity for each conductive yarn and stitching density. Finally, a comparison of conductive yarn usage in different embroidering scenarios is presented. These analyses provide valuable insights into the impact of stitching density and yarn properties on the overall performance of embroidered TLs.

### 4.1. Single Stitching

In this subsection, we present the losses (L) results for single stitching for both yarns (Shieldex and Elitex) for all examined stitch densities using our TL technique. For comparison and validation reasons, we use the measured results of Elitex for all stitch densities, and we have produced the FLF graph. As mentioned, explained, and analyzed in the introduction section, FLF is very good for stable soldered TLs, but is not appropriate for removable TLs, such as ours.

A. Proposed technique (mismatch losses exclusion)

The losses of the TLs, where the density pattern is stitched only once, are illustrated in the following graphs across the 0.5–4.0 GHz frequency range, utilizing the Shieldex and Elitex yarns, respectively.

**Figure 8:**
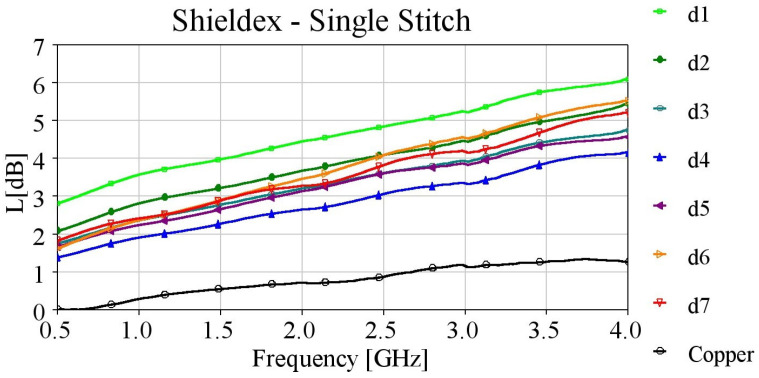
Measured loss (L[dB]) of 10 cm-long Shieldex-embroidered microstrip samples with single stitching.

Microstrips embroidered with single stitching using the Shieldex yarn demonstrate considerably higher losses across the entire frequency spectrum in comparison to the copper microstrip line (Figure 8). Enhanced performance is observed with increased stitch density up to 4 lines/mm. However, further increasing the stitch density results in higher losses for the embroidered samples, despite their robust conductive patterns, which is attributed to the CFC feeding method. Consequently, sample d4 demonstrates superior performance, with losses of almost 3 dB at 2.5 GHz.

**Figure 9:**
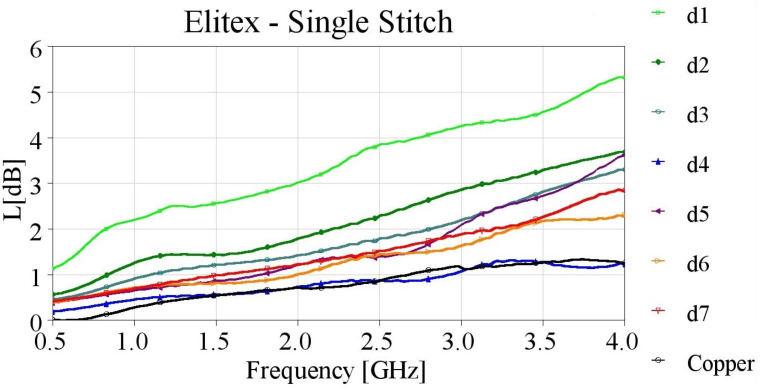
Measured loss (L[dB]) of 10 cm-long Elitex-embroidered microstrip samples with single stitching.

Microstrip, embroidered with single stitching using the highly conductive Elitex yarn outperformed those with the Shieldex yarn. As displayed in Figure 9, their losses were consistently below 3 dB, even for the lowest stitch densities. Once again, the d4 sample presented the best performance, with losses strikingly close to the losses measured for the reference copper microstrip, across the entire examined frequency band. Hence, we successfully produced an embroidered microstrip line that exhibits a performance comparable to the reference copper line, achieved through a moderately high stitch density (4 lines/mm).

The Shieldex yarn stood out for its “embroiderability”, due to its smooth, flexible threads, which minimized the duration of the embroidery process, with minor disruptions. Nevertheless, its filaments’ thin metallization resulted in lossy embroidered lines. On the other hand, the inherent high conductivity of the Elitex yarn led to the production of low-loss embroidered TLs. However, the stiffness of the conductive yarn introduced high tension during embroidery, leading to occasional thread breakage and the formation of a rough, textured conductive surface.

**Figure 10:**
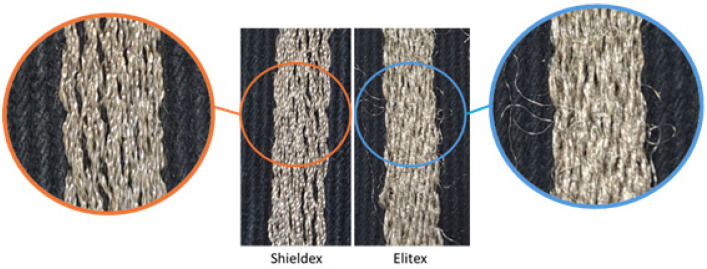
Close-ups of sample d5 embroidered with Shieldex and Elitex yarn, respectively.

Figure 10 displays a close-up photo of two samples with density equal to 5 lines/mm embroidered with Shieldex and Elitex yarns, respectively. Contrary to the clean-cut Shieldex embroidery, several broken filaments are visible on the Elitex yarn sample, on top of its rough conductive surface. The breakage of threads became more prominent as stitch density increased, which could be attributed to the high frequency introduced by the closely stitched yarns. According to [60], broken filaments result in an increase in the conductor loss of the conductive embroidery. Therefore, excessive thread breakage at higher frequencies could be the reason why increasing the stitch density does not have a significant effect on the performance of the embroidered samples employing the Elitex yarn.

For both yarns, it has been noticed that above 4 lines/mm stitch density, the performance starts to degrade, and not very stable results occur. This can be attributed to the inhomogeneity introduced at the interface point with Shieldit, caused by the increase in holes due to the higher stitch density and dense pitches.

While the Elitex yarn may be the preferred choice for low-loss microstrip TLs, it is crucial to consider its higher cost, as well as the increased complexity of the embroidery process caused by their stiff conductive filaments.

B. Comparison of the proposed technique with Forward Loss Factor (FLF) technique

**Figure 11:**
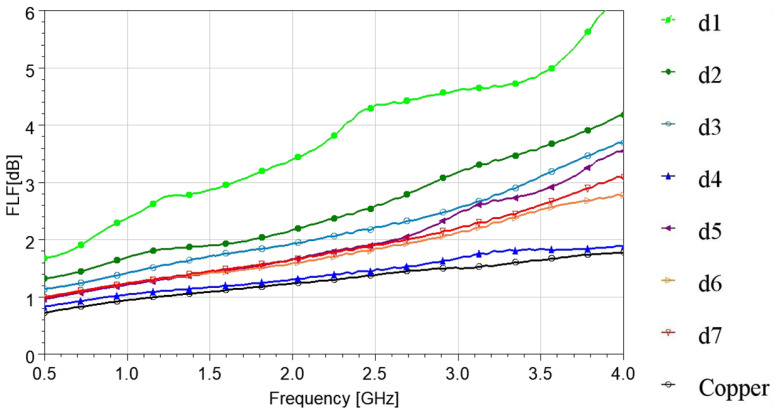
Measured forward loss factor (FLF [dB]) of 10 cm-long Elitex-embroidered microstrip samples with single stitching.

For comparison reasons with other TL techniques applied in the literature, we have produced the FLF results [32] for the Elitex measurements (Figure 11). If we compare Figure 9 and Figure 11, similar conclusions can be drawn. The d4 sample presented the best performance, with losses close to the losses measured for the reference copper microstrip across the entire examined frequency band. Hence, we successfully produced an embroidered microstrip line that exhibits a performance comparable to the reference copper line, achieved through a moderately high stitch density (4 lines/mm). Generally, the loss comparison between all stitch densities is similar, as in the previous subsection, which leads to similar conclusions for the Elitex single-stitch yarn. It must be noted that the FLF losses are higher than the L (our technique) losses. This is due to the exclusion of all mismatch losses (and S22) in our technique. This is the reason that our technique is more appropriate for removable embroidered, non-soldered patterns, where the S11 and S22 can vary and must, therefore, be excluded from the losses calculation.

### 4.2. Double Stitching

The following graphs display the losses of the double stitched embroidered microstrip lines using the studied conductive yarns correspondingly. The measured results of the copper line are also displayed as reference.

**Figure 12:**
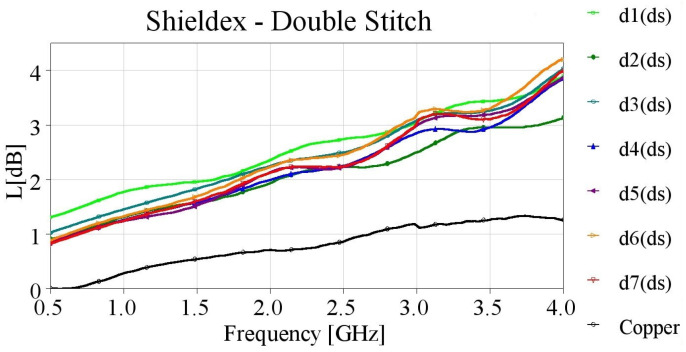
Measured loss (L[dB]) of 10 cm-long Shieldex-embroidered microstrip samples with double stitching.

The Shieldex yarn demonstrated enhanced performance in terms of losses when double stitching was applied to the embroidered microstrips. Notably, the losses of all samples remained below 4 dB (Figure 12) in the majority of the examined frequency spectrum. However, all TLs demonstrated comparable performance across the entire studied spectrum, regardless of the stitch density. Consequently, the use of double stitching presents satisfactory performance even at lower stitch densities.

**Figure 13:**
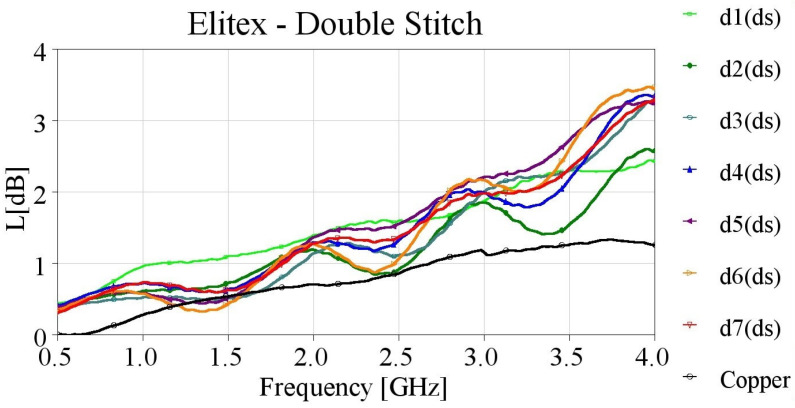
Measured loss (L[dB]) of 10 cm-long Elitex-embroidered microstrip samples with double stitching.

As seen in Figure 13, double-stitched samples with the Elitex yarn also exhibited comparable losses, especially at frequencies up to 3.0 GHz. Notably, among the double stitched samples, d2 (ds) presented the best performance across the entire frequency range. Additionally, while the single stitched d4 sample exhibited losses close to copper, the double-stitched counterpart, d4 (ds), presented a weaker performance. Lastly, unlike the Shieldex yarn, Elitex yarn double stitching did not have a profound effect on the microstrip lines’ performance compared to single stitching.

### 4.3. Repeatability of Fabrication and Measurements

The upcoming paragraphs will focus on repeatability measurements related to the fabrication of embroidered samples. Furthermore, the repeatability of the measuring process will be discussed to assess the reliability of measured results and to provide meaningful observations concerning the stability of the performance of the embroidered prototypes.

Repeatability measurements were conducted between duplicate prototypes (di_a and di_b, i = 1, 2, …, 7) to assess the variation in microstrip lines’ performance due to fabrication discrepancies. These measurements offer valuable insights into the feasibility of utilizing embroidery techniques for the mass production of wearable RF components. 

**Figure 14:**
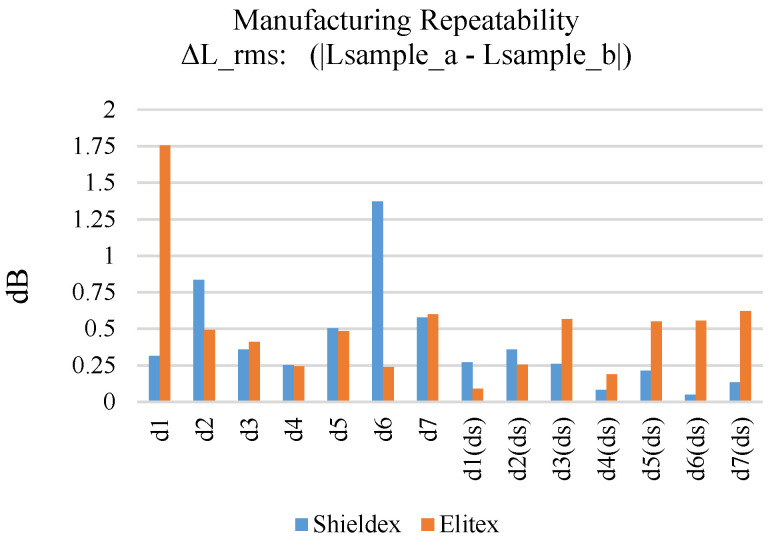
Manufacturing repeatability (a–b samples comparison) of Shieldex- and Elitex-embroidered microstrip samples.

Figure 14 displays the RMS difference (ΔL_rms) in the loss along the entire frequency range (1601 sample points), between prototypes a and b for each embroidered line sample (single and double stitching).

Generally, double stitching samples embroidered with the Shieldex yarn exhibit improved repeatability, as the calculated ΔL_rms did not exceed 0.6 dB for any sample. On the other hand, samples embroidered with single stitching displayed ΔL_rms values between 0.25 dB and 0.8 dB, except for sample d6 whose ΔL_rms reached 1.3 dB. The samples embroidered with the Elitex yarn generally exhibited higher ΔL_rms values compared to the Shieldex yarn. It is also noted that double stitching did not have a noticeable effect on manufacturing stability for the Elitex-embroidered samples, contrary to the Shieldex samples. Thread breakage due to the higher friction developed between the Elitex stitches may be responsible for significant structural discrepancies between the a and b samples.

**Figure 15:**
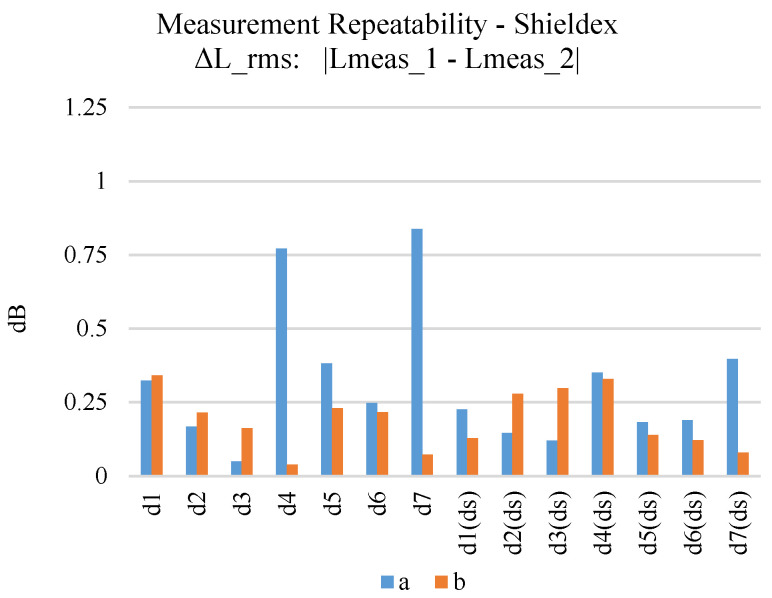
Measurement repeatability (first–second measurement comparison) of Shieldex-embroidered microstrip samples.

**Figure 16:**
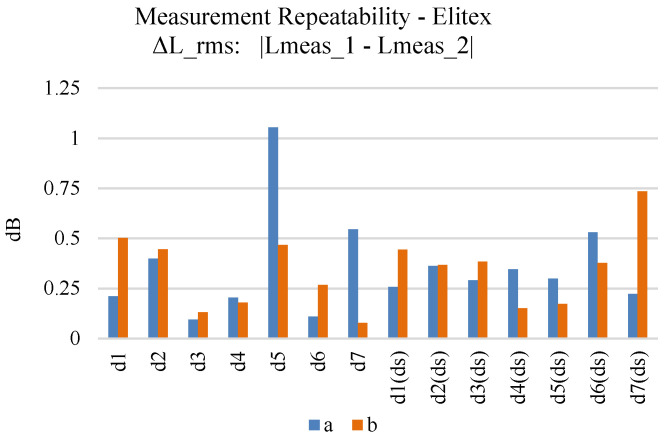
Measurement repeatability (first–second measurement comparison) of Elitex-embroidered microstrip samples.

In order to improve the uncertainty for the derived loss measurement results, all individual prototypes were measured twice, at different timelines. Figure 15 displays the measuring repeatability, i.e., the difference between the two measurements of each sample, for the samples embroidered using the Shieldex yarn. Double stitching has a positive effect on the measuring repeatability of the microstrips, providing an average of 0.3 dB RMS difference. Still, even measurements of single stitching samples present an acceptable repeatability (around 0.25 dB RMS difference) for most of the samples. The measurement repeatability of the Elitex yarn can be seen in Figure 16. Double stitching provided a 0.3 dB RMS difference. Still, the observed measurement deviations did not exceed 0.5 dB RMS difference for the majority of the Elitex single stitching samples. In general, samples embroidered using the Shieldex yarn displayed better measurement repeatability compared to the Elitex-embroidered samples.

Therefore, the utilization of the designed measurement jig facilitated the measurement of a large number of embroidered microstrip line samples on a common substrate. With no soldering and a simple pressure connection to the VNA, a repeatability study verified measurement reliability, with repeatable measurements exhibiting variations of less than 0.5 dB in most cases.

### 4.4. Effective Conductivity

Unlike conventional microstrip TLs, the upper conductor in our embroidered microstrips is not a rigid metal with uniform conductivity [61]; rather, it is composed of conductive yarns embroidered onto denim fabric. To evaluate the microstrip lines’ performance, we have introduced the concept of the effective conductivity of each microstrip [62]. This refers to the conductivity value that a conventional TL exhibiting equivalent losses (L[dB]) would have, providing that the same base fabric and substrate were used.

The effective conductivity for each sample was determined through parametric EM simulations (using Ansys HFSS) and comparison with the corresponding loss (L[dB]) measurement results. This process involved selecting the most suitable loss curve by evaluating the difference (RMS difference) between the measured and simulated curves across the entire frequency spectrum from 0.5 to 4.0 GHz.

**Figure 17:**
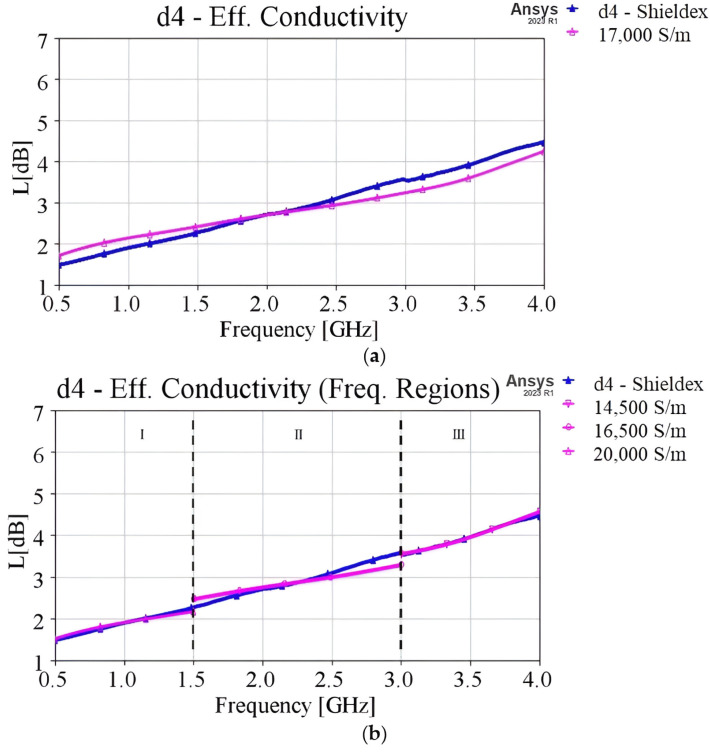
(**a**) Initial estimation of the effective conductivity of sample d4 (Shieldex), throughout the whole frequency range (0.5–4.0 GHz), and (**b**) estimation of the effective conductivity individually for each frequency region.

To demonstrate this process using a specific yarn and stitch density, in Figure 17, the measured loss curve of the Shieldex d4 sample is displayed along with the simulated curve corresponding to the initial approximation of conductivity 17 × 10^3^ S/m. The initial approximation (Figure 17a) revealed the frequency dependence (dispersion) of effective conductivity, especially noticeable at lower and higher frequencies. To address this, we subdivided the frequency spectrum into three regions: (I) 0.5–1.5 GHz, (II) 1.5–3.0 GHz, and (III) 3.0–4.0 GHz, re-evaluating the effective conductivity for each region. At lower frequencies (0.5–1.5 GHz), an effective conductivity of 14.5 × 10^3^ S/m provided the best fit to the measured curve, reducing the RMS difference from 0.232 dB to 0.017 dB compared to the initial approximation. For region II (1.5–3.0 GHz), the estimated effective conductivity was 16.5 × 10^3^ S/m, resulting in a slight change in RMS difference from 0.037 dB to 0.033 dB. Finally, at higher frequencies (3.0–4.0 GHz), an improved effective conductivity approximation of 14.5 × 10^3^ S/m was derived, significantly reducing the RMS difference from 0.303 dB to 0.027 dB. In Figure 17b, it may seem that in region II, there is an observable difference between L measured and estimated curves. However, it should be noted that the RMS difference decreased compared to the initial estimation, as it went down to 0.033 dB. This RMS error is the largest compared with other frequency regions, but it remains quite small, giving the optimum estimation of the actual losses for the specific yarn (Shieldex) and density (d4). It should be noted that such observable differences can be seen in other frequency regions for other samples. The main criterion for the estimation of effective conductivity was that the RMS difference will be below 0.1 dB.

Therefore, by estimating the effective conductivity for each region individually, we can achieve better curve fitting for each frequency region compared to the estimation that considers the whole frequency range. This approach facilitated the development of an equivalent EM model for each sample, which accurately represents its losses.

The following table (Table 3) shows the estimated values of the effective conductivity of each of the samples, embroidered with single stitching for both examined yarns.

**Figure 18:**
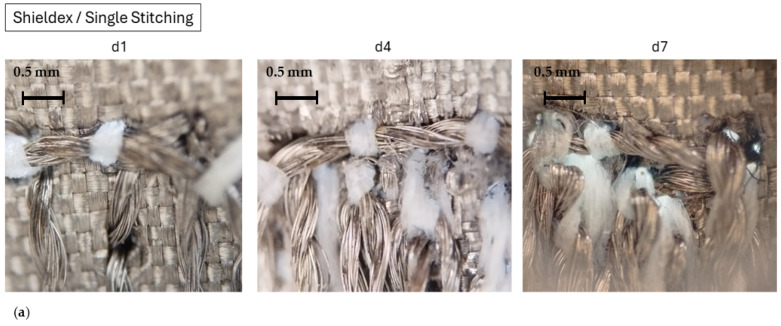

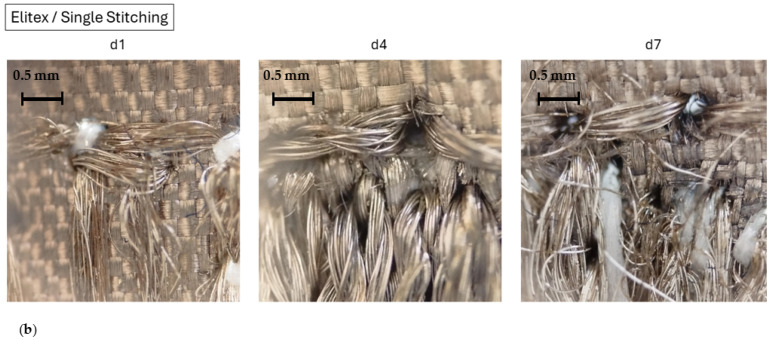
Microscope zoomed-in photos of single-stitch embroidered patterns on the interface point for densities d1, d4, d7 for (**a**) Shieldex and (**b**) Elitex yarns.

In general, samples embroidered with Elitex yarn exhibited notably higher effective conductivity, which in most cases surpassed twice the conductivity observed when using Shieldex yarn. It is noted that the d4 samples demonstrate the highest effective conductivity for both Shieldex and Elitex yarns. At this density, the derived effective conductivity of Elitex yarn samples approached that of copper (58,000 × 10^3^ S/m). At this density (d4), the Elitex single stitch pattern probably results in a very smooth and suitably dense pattern with minimum needle puncture damage to the conductive cloth interface part, ensuring high conductivity and uniform current distribution (Figure 18b). In fact, both yarns yielded samples with higher effective conductivity as the stitch density increased, reaching its peak at density 4 lines/mm (d4). However, with further stitch density increases, the samples presented increased losses, leading to a decrease in estimated effective conductivity. This rise in losses was attributed to the feeding technique, suggesting that the conductive fabric used for interconnecting the embroidered lines to the SMA connectors (CFC feeding) might suffer damage from multiple needle punches, resulting in degraded microstrip line performance. This last point is clearly visible in Figure 18.

Utilizing the double stitching technique enhanced the effective conductivity of all Shieldex yarn-embroidered samples (Table 4). Likewise, the double-stitched Elitex samples exhibited improved performance, with the exception of sample d4 (ds) which displayed lower effective conductivity compared to the single stitching case. Nonetheless, both yarns showed lower effective conductivity at densities of 5 to 7 lines/mm, once again attributed to the feeding technique. Lastly, it was observed that while employing the Elitex yarn was favorable to increasing effective conductivity, this effect was generally less pronounced at higher frequencies. For instance, the effective conductivity in the lowest frequency region of the Elitex yarn sample d1 (ds) is 226% higher compared to the Shieldex yarn. This percentage falls to 176% for the same samples in the highest frequency region. Likewise, d3 (ds) drops from 1900% to 100%, and d5 (ds) experiences a decrease from 757% to 58%.

### 4.5. Conductive Yarn Usage Comparison

As far as practical applications of embroidered textile electronics are concerned, in addition to ΕΜ performance, the cost of fabrication also plays a vital role. In our study, the performance of the embroidered microstrip lines has been investigated through their effective conductivity. The fabrication cost is influenced by the type of conductive yarn used and the corresponding amount of yarn usage for the selected stitch density.

In order to obtain insight into the trade-off between the performance and conductive yarn usage of embroidered microstrip lines, we calculated an estimate of the total yarn used (length in m) per sample. The total length of yarn needed to form one stitch was estimated as the stitch length plus two times the base fabric thickness, which corresponds to the extra yarn used to form the lock stitch [60,63]. The total yarn usage was subsequently calculated by multiplying the yarn used to form one stitch by the total stitch number per sample (Table 5).

The yarn usage can provide essential information on the cost effectiveness of the embroidered samples, which plays a crucial role, especially in the prospect of mass production [64]. For example, roughly 2.58 m of yarn is required to fabricate a sample with a density equal to 2 lines/mm. By using approximately 64% more yarn, we can fabricate a d2 (ds) sample, which in the case of the Shieldex yarn would provide decreased losses, as well as improved manufacturing and performance repeatability [65]. Using almost the same amount of yarn (4.18 m), we could also fabricate a d4 sample with the more expensive Elitex yarn, whose performance would be comparable to a copper microstrip line.

## 5. Feeding Techniques Comparison (CFC vs. YC)

During the course of our study, it was observed that contrary to the bibliography [16], increasing the stitch density of the embroideries did not always have a positive effect on the performance of the embroidered lines. Specifically, a majority of the samples exhibited the best performance at a density of 4 lines/mm, and further density increments resulted in a decrease in effective conductivity. This phenomenon was attributed to the feeding method (CFC feeding), which involved integrating a small segment of conductive fabric at the open ends of the microstrips. While the initial intent behind this feeding technique was to find a more realistic scenario and to ensure proper connection of the embroidered TL to the SMA connectors’ pins, it proved advantageous for sparser stitches (≤4 lines/mm) but led to a notable increase in losses at higher stitch densities.

**Figure 19:**
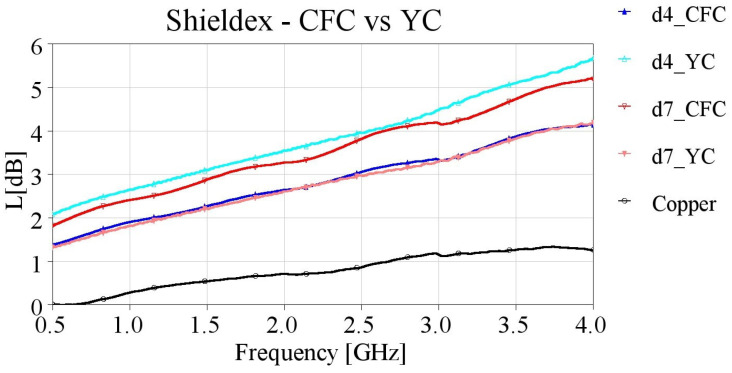
Measured loss (L[dB]) of 10 cm-long Shieldex-embroidered microstrip samples with single stitching with densities equal to 4 and 7 lines/mm, employing CFC and YC feeding.

**Figure 20:**
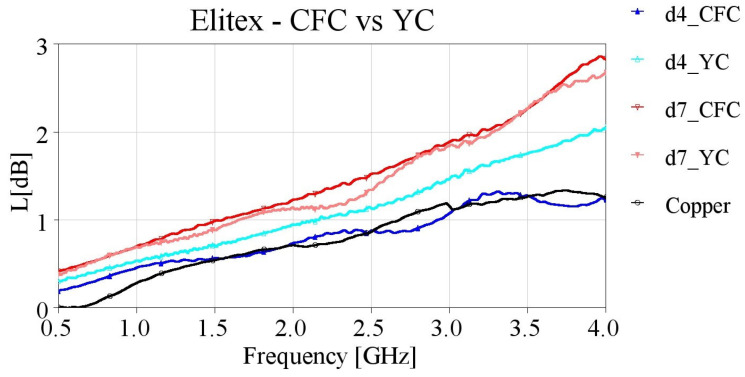
Measured loss (L[dB]) of 10 cm-long Elitex-embroidered microstrip samples with single stitching with densities equal to 4 and 7 lines/mm, employing CFC and YC feeding.

**Figure 21:**
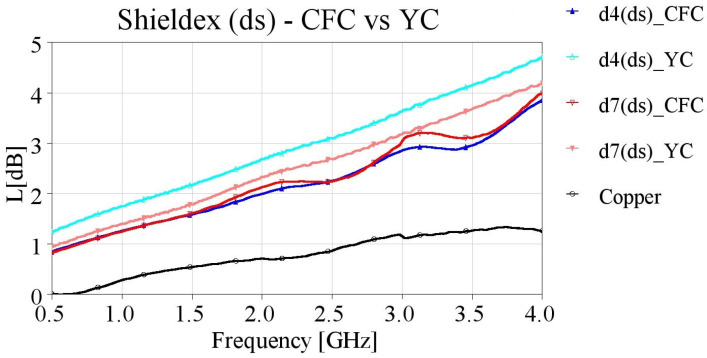
Measured loss (L[dB]) of 10 cm-long Shieldex-embroidered microstrip samples with double stitching for densities equal to 4 and 7 lines/mm, employing CFC and YC feeding.

**Figure 22:**
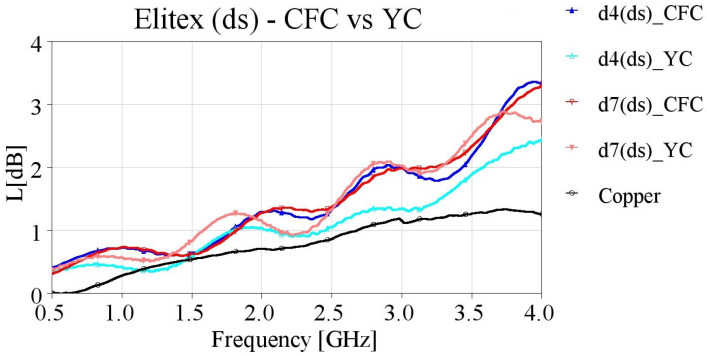
Measured loss (L[dB]) of 10 cm-long Elitex-embroidered microstrip samples with double stitching for densities equal to 4 and 7 lines/mm, employing CFC and YC feeding.

In order to verify this assumption, we created additional samples with direct contact from the SMA connectors’ pins to the embroidered yarn (YC feeding), meaning that the entire length of the microstrips was embroidered. The YC feeding method was implemented for embroidering samples with densities of 4 and 7 lines/mm. The measured results of the yarn contact samples are presented in Figure 19, Figure 20, Figure 21 and Figure 22, along with the CFC samples for the two studied densities, with single and double stitching and for both yarns.

Microstrips embroidered with Shieldex yarn and utilizing YC feeding demonstrate opposite results in terms of total measured losses compared to the YC samples (Figure 19). Specifically, the d4_YC sample exhibits degraded performance with increased losses of approximately 1 dB at 2.5 GHz compared to the d4 CFC sample (d4_CFC). This observation was expected, since CFC feeding was initially designed to aid the connection to the SMA connectors and is crucial, especially for low density samples.

On the other hand, by omitting the conductive fabric at the feeding points, the d7_YC sample shows improved performance, with a decrease of 1 dB in losses compared to the d7_CFC sample. The improvement of the d7 sample’s performance when YC is applied reinforces the consideration that in higher density samples, the large number of closely located needle punches damages the conductive fabric, therefore impairing connectivity and increasing losses. Moreover, the densely stitched adjacent conductive yarns assure proper current distribution on the entire width of the microstrip; thus, employing the CFC feeding is not necessary.

In contrast to the Shieldex microstrips, YC feeding had minimal impact on the losses of Elitex samples, as seen in Figure 20. Thus, with Elitex yarn, increasing the stitch density does not consistently guarantee improved effective conductivity, as seen in Table 6 and Table 7. Additionally, it should be noted that utilizing a relatively sparse embroidery pattern, can yield embroidered microstrips with a performance comparable to a copper solid microstrip line while keeping the material usage and, therefore, the total manufacturing cost low. Moreover, using the highly conductive Elitex yarn resulted in embroidered microstrip lines with significantly improved performance compared to the microstrips embroidered using the Shieldex yarn.

As seen in Table 6, the effective conductivity of the d4_YC sample significantly decreased for both conductive yarns when YC feeding was employed, as a result of poor connection of the SMA connectors to the sparse conductive stitches. On the contrary, YC improved the effective conductivity of samples with a density of 7 lines/mm for both yarns (Table 7), since using conductive fabric (CFC) at the open ends of the microstrips degraded their performance. Consequently, when YC feeding was applied, increasing the stitch density had a favorable result on the effective conductivity of the embroidered microstrips.

The impact of YC feeding was also investigated in the case of double stitching. When Shieldex yarn was employed, similarly to single stitched samples, the d4 (ds)_YC sample exhibited higher losses compared to d4 (ds)_CFC. Additionally, the performance of double-stitched microstrips with a density of 7 lines/mm slightly improved with the YC feeding method (Figure 21).

Figure 22 displays the measured results of the Elitex yarn-embroidered samples with double stitching, employing YC and CFC feeding. The d4 (ds)_YC sample exhibited outstanding performance, with losses comparable to that of the reference copper microstrips, especially for frequencies up to 3.0 GHz. In the case of a stitch density of 7 lines/mm, employing YC had a negligible effect on the losses of the microstrip lines, compared to the CFC samples.

As far as the effective conductivity of the double-stitched samples is concerned, increasing the stitch density has favorable effect on the samples embroidered with the Shieldex yarn. More specifically, at 2.5 GHz, the σeff increased from 16.5 × 10^3^ S/m to 21 × 10^3^ S/m when stitch density increased from 4 to 7 lines/mm, respectively (Table 8 and Table 9).

The effective conductivity of sample d4 (ds)_YC, embroidered with the Elitex yarn, reached 10^6^ S/m at 2.5 GHz (Table 8), compared to 120 × 10^3^ S/m which is the estimated effective conductivity of the sample with a density equal to 7 lines/mm (Table 9). Therefore, in this case increasing the stich density did not improve the performance of the microstrips. As discussed earlier, this effect may be attributed to the excessive thread snapping of the Elitex yarn (Figure 10 and Figure 18), caused by the high tensions developed due to its rough, stiff surface.

## 6. Conclusions

In this paper, a new measurement technique for evaluating textile transmission lines with different embroidery characteristics (patterns, yarns, stitching, etc.) has been proposed. The evaluation metric parameter used by this technique is the losses (pure losses) of the embroidered TL, excluding the mismatch losses at the ports (in/out) of the line. The repeatability of the measurement results has been evaluated with satisfactory results (about 0.5 dB RMS deviation) for all examined embroidered patterns. Also, important hints about the digitization and fabrication of the embroidery pattern, such as to adjust pattern dimensions by considering the yarn thickness and the settings of the embroidery machine stitching speed, are given. The fabrication repeatability has also been examined and evaluated via the TL loss measurements, with derived deviations of around 0.7 dB.

Also, via measurements, a comprehensive exploration of embroidered microstrip TLs, focusing on the impact of stitch density and conductive yarn type on their performance has been conducted. This led to the derivation of practical insights that could significantly contribute to the mass development of embroidered TLs, as well as antennas, and other textile electromagnetic components. More specifically, two conductive yarns (Shieldex: low conductivity, Elitex: high conductivity) were studied with varying embroidery stitch density and stitch pattern (i.e., single/double stitching). Increasing the stitch density of the embroidery design enhanced the performance of the embroidered microstrips up to density 4 lines/mm. When the stitch density exceeded 5 lines/mm, additional losses were introduced by the CFC feeding technique. Therefore, while CFC feeding is suitable for low-stich-density designs, employing YC feeding is more suitable for high-stitch-density embroidery. Double stitching had a favorable effect on the performance of the microstrip lines embroidered with Shieldex yarn, in terms of losses, as well as manufacturing repeatability. Employing double stitching on the samples embroidered with the Elitex yarn did not significantly improve their performance, especially in higher stich densities, which could be attributed to extensive thread breakage. Last but not least, the microstrip line embroidered with Elitex yarn and single stitching with density equal to 4 lines/mm exhibited remarkably low losses, comparable to the reference copper microstrip- when CFC feeding was employed.

Hence, contrary to the prevailing assumption in the literature that increasing stitch density enhances the performance of embroidered electromagnetic components, this experimental study reveals that this is not always applicable. This variance is dependent upon factors, such as stitch pattern (single/double stitching), feeding method, and the properties of the conductive yarn employed. Consequently, a thorough examination of the performance of the selected conductive yarn is imperative, while also taking into account the “embroiderability” and total material cost. It has been found that using more yarn will not always provide better pattern performance. It is important to know the effects of yarn usage, so as to make specific trade-offs between performance and cost.

Finally, the conductivity of each sample has been electromagnetically examined via simulations by using the loss measurements data. It has been found, that for all samples the effective conductivity is frequency-dependent, and that it rapidly changes, above 3.0 GHz.

## Figures and Tables

**Table 1:** Examined samples characteristics: name, density, and number of total stitches.

Sample Name	Density [lines/mm]	Total Stitches
d1	1	315
d2	2	410
d3	3	509
d4	4	706
d5	5	803
d6	6	900
d7	7	1000
d1 (ds)	1 (ds)	527
d2 (ds)	2 (ds)	717
d3 (ds)	3 (ds)	915
d4 (ds)	4 (ds)	1313
d5 (ds)	5 (ds)	1503
d6 (ds)	6 (ds)	1697
d7 (ds)	7 (ds)	1897

**Table 2:** Specifications of conductive yarns.

Yarn	Yield [m/kg]	Dtex	# of Plies	# of Filaments	Linear Resistance [Ω/m]
Shieldex	35,000	117	2	34 (17 × 2)	3000
Elitex	22,000	235	1	34	20

**Table 3:** Effective conductivity of samples embroidered with single stitching for both examined yarns.

Sample	0.5–1.5 GHz	1.5–3.0 GHz	3.0–4.0 GHz	0.5–1.5 GHz	1.5–3.0 GHz	3.0–4.0 GHz
	Shieldex σ_eff_ [(S/m) × 10^3^]			Elitex σ_eff_ [(S/m) × 10^3^]		
d1	8.5	8.0	7.5	18.0	13.0	10.0
d2	12.0	11.0	9.0	47.0	31.0	19.0
d3	15.0	12.5	11.5	75.0	47.0	28.0
d4	20.0	16.5	14.5	5000.0	41,000.0	41,000.0
d5	16.0	12.5	12.0	140.0	70.0	28.0
d6	15.0	10.5	9.0	110.0	140.0	45.0
d7	14.0	11.0	10.0	120.0	70.0	45.0

**Table 4:** Effective conductivity of samples embroidered with double stitching for both examined yarns.

Sample	0.5–1.5 GHz	1.5–3.0 GHz	3.0–4.0 GHz	0.5–1.5 GHz	1.5–3.0 GHz	3.0–4.0 GHz
	Shieldex σ_eff_ [(S/m) × 10^3^]			Elitex σ_eff_ [(S/m) ) × 10^3^]		
d1 (ds)	23.0	20.5	17.0	75.0	60.0	47.0
d2 (ds)	36.0	30.0	24.0	180.0	200.0	140.0
d3 (ds)	30.0	22.0	18.0	600.0	200.0	36.0
d4 (ds)	40.0	27.5	21.0	140.0	100.0	40.0
d5 (ds)	35.0	25.0	19.0	300.0	90.0	30.0
d6 (ds)	36.0	23.0	17.0	500.0	90.0	65.0
d7 (ds)	39.0	25.0	19.0	200.0	100.0	40.0

**Table 5:** Total yarn used (length in m) per sample.

Sample	Yarn Usage [m]	Sample	Yarn Usage [m]
d1	2.07	d1 (ds)	3.21
d2	2.58	d2 (ds)	4.24
d3	3.12	d3 (ds)	5.31
d4	4.18	d4 (ds)	7.46
d5	4.70	d5 (ds)	8.48
d6	5.23	d6 (ds)	9.53
d7	5.77	d7 (ds)	10.61

**Table 6:** d4: Effective conductivity of CFC and YC samples.

Sample	0.5–1.5 GHz	1.5–3.0 GHz	3.0–4.0 GHz	0.5–1.5 GHz	1.5–3.0 GHz	3.0–4.0 GHz
	Shieldex σ_eff_ [(S/m) × 10^3^]			Elitex σ_eff_ [(S/m) × 10^3^]		
d4_CFC	20.0	16.5	14.5	5000.0	41,000.0	41,000.0
d4_YC	12.5	11.0	9.0	300.0	300.0	200.0
$\frac{CFC-YC}{CFC}$	−37.5%	−33.3%	−37.9%	−94.0%	−99.3%	−99.5%

**Table 7:** d7: Effective conductivity of CFC and YC samples.

Sample	0.5–1.5 GHz	1.5–3.0 GHz	3.0–4.0 GHz	0.5–1.5 GHz	1.5–3.0 GHz	3.0–4.0 GHz
	Shieldex σ_eff_ [(S/m) × 10^3^]			Elitex σ_eff_ [(S/m) × 10^3^]		
d7_CFC	14.0	11.0	10.0	120.0	70.0	45.0
d7_YC	22.0	17.5	14.5	130.0	110.0	200.0
$\frac{CFC-YC}{CFC}$	57.1%	59.1%	45.0%	8.3%	57.1%	344.4%

**Table 8:** d4 (ds): Effective conductivity of CFC and YC samples.

Sample	0.5–1.5 GHz	1.5–3.0 GHz	3.0–4.0 GHz	0.5–1.5 GHz	1.5–3.0 GHz	3.0–4.0 GHz
	Shieldex σ_eff_ [(S/m) × 10^3^]			Elitex σ_eff_ [(S/m) × 10^3^]		
d4 (ds)_CFC	40.0	27.5	21.0	140.0	100.0	40.0
d4 (ds)_YC	23.0	16.5	12.5	1000.0	1000.0	390.0
$\frac{CFC-YC}{CFC}$	−42.5%	−40.0%	−40.5%	614.3%	900.0%	875.0%

**Table 9:** d7 (ds): Effective conductivity of CFC and YC samples.

Sample	0.5–1.5 GHz	1.5–3.0 GHz	3.0–4.0 GHz	0.5–1.5 GHz	1.5–3.0 GHz	3.0–4.0 GHz
	Shieldex σ_eff_ [(S/m) × 10^3^]			Elitex σ_eff_ [(S/m) × 10^3^]		
d7 (ds)_CFC	39.0	25.0	19.0	200.0	100.0	40.0
d7 (ds)_YC	32.0	21.0	16.5	200.0	120.0	120.0
$\frac{CFC-YC}{CFC}$	−17.9%	−16.0%	−13.2%	0.0%	20.0%	200.0%

## Data Availability

Data are contained within the article.
